# Ecology, genetics and distribution of *Punctoribates zachvatkini*, an oribatid mite so far overlooked in Germany

**DOI:** 10.1007/s10493-022-00738-3

**Published:** 2022-08-08

**Authors:** Julian Escher, Karin Hohberg, Peter Decker, Ricarda Lehmitz

**Affiliations:** 1 Senckenberg Museum of Natural History Görlitz, Am Museum 1, 02826 Görlitz, Germany; 2 Blumenstr. 5, 02826 Görlitz, Germany

**Keywords:** Octotaxic system, Species separation, Habitat preference, Nuclear 28S rDNA, Museum collections

## Abstract

*Punctoribates* is one of few genera in Poronota (Acari: Oribatida) containing species with porose areas and species with saccules, the two types of the octotaxic system. These porose organs are the main difference between two morphologically similar species, *P. punctum* with porose areas and *P. zachvatkini* with saccules. As the octotaxic system can vary within species, species separation solely based on this trait might be insufficient. To assess the species status of *P. zachvatkini*, we investigated additional differences from *P. punctum* by comparing habitat preferences of the two species regarding nature reserves and agricultural landscapes during a field study in the German Eifel region, and by examining *Punctoribates* material from four large German natural history museums. We also performed scanning electron microscopy (SEM) and a genetic analysis using the D3 marker of the nuclear 28S rDNA gene. In the field study, *P. zachvatkini* had higher densities in the nature reserves and *P. punctum* in the agricultural landscapes. Evaluation of the museum material revealed *P. punctum* occurred more regularly in disturbed sites such as urban, agricultural and post-mining areas compared to *P. zachvatkini*. Pairwise distances of the 28S D3 genetic marker as well as an additional base pair in *P. zachvatkini* further support the separation of the two species, and SEM investigations revealed new details regarding the punctulation of *P. zachvatkini*. The review of the museum material showed that *P. zachvatkini* already occurred in Germany in 1967 and has a wider distribution than previously known.

## Introduction

*Punctoribates*, a member of the family Punctoribatidae (junior synonym Mycobatidae; see Subías 2004 for discussion), is a globally distributed genus of Poronota (Acari: Oribatida) with currently 30 known species (Seniczak et al. 2020). It is one of few genera within the Poronota to contain species with porose areas as well as species with saccules, the two regular types of the octotaxic system. The octotaxic system is a set of usually four pairs of porose organs on the notogaster with secretory function (Alberti et al. 1996). In most Poronota, the octotaxic system is expressed as either porose areas (exposed pore fields on the cuticle) or saccules (invaginated pockets of pores beneath the cuticle).

Most species in *Punctoribates* express porose areas. Among them is *Punctoribates punctum* (Koch), the globally distributed type species of the genus, which is present throughout most of Germany (Weigmann et al. 2015). A species with saccules is *Punctoribates zachvatkini* (Shaldybina), which has been mostly recorded in Eastern Europe and Russia (Shaldybina 1969; Ivan and Călugăr 2004; Shevchenko and Kolodochka 2014). The first findings of *P. zachvatkini* in the German federal states of Saxony and Bavaria (Weigmann et al. 2015) represent the most western record of the species currently known.

For *Punctoribates* species with saccules, the genus *Semipunctoribates* has been proposed (Mahunka 1987; Bayartogtokh et al. 2000). However, there is discussion as to whether the octotaxic system suffices to split up a genus (Weigmann 2010; Weigmann and Ermilov 2016; Seniczak et al. 2020). Although the occurrence of both types of the octotaxic system in the same genus is rare, it can vary between genera of a family (Alberti and Norton 1997; Schäffer et al. 2010). This suggests a plasticity of the trait, possibly linked to functional selection (Schäffer et al. 2010; Klimov and Ermilov 2017), although the advantage of either an exposed or invaginated secretory surface is not clear (Alberti and Norton 1997). In a few cases, variability is known to extend to the level of populations or even individuals: multiple specimens in a population of *Protoribates paracapucinus* (Mahunka) (Haplozetidae) expressed saccules instead of porose areas (Weigmann and Ermilov 2016), and a specimen of *Peloptulus phaeonotus* (Koch) (Phenopelopidae) had one porose area replaced by a saccule (Weigmann 2010). This raises the question of how reliable the type of octotaxic system is for species separation.

Morphologically, *P. zachvatkini* is very similar to *P. punctum*, being smaller on average, but their size ranges overlap. In addition, there are slight differences in the shape of bothridial and length of notogastral setae (Seniczak et al. 2020). However, the most notable distinction is their octotaxic system. The slight morphological differences and possible plasticity of the octotaxic system lead to the question of whether *P. zachvatkini* and *P. punctum* are indeed two separate species or just represent intraspecific variation within *P. punctum*. Due to their morphological similarity and as *P. zachvatkini* is not included in the most recent identification key for Central European Oribatida, published by Weigmann (2006), *P. zachvatkini* may have been confused with *P. punctum* in the past. The actual distribution of the two species and their ecological requirements are therefore not known, nor is whether they are truly two species.

A combined approach including genetics and ecology is known to reliably separate even cryptic species (Rissler and Apodaca 2007; Padial et al. 2010; Fišer et al. 2018). Closely related, sympatric species often differ in habitat use (Reinert 1984; Friberg et al. 2008), meaning that information about habitat preferences can provide arguments regarding species status. Furthermore, genetic markers have become a standard method to test separation of species. For Oribatida, the nuclear 28S D3 region is a reliable marker for this purpose (Maraun et al. 2004; Lehmitz and Decker 2017).

The aim of the present study is to clarify the morphologically ambiguous separation of *P. zachvatkini* and *P. punctum*. Therefore, we investigated habitat preferences in a field study in the Eifel region in Germany, where both species occurred sympatrically, and we reviewed *Punctoribates-*collection material from four large German natural history museums, in order to gather all available information on the distribution of the possibly overlooked *P. zachvatkini* in Germany. We hypothesized (1) that *P. punctum* and *P. zachvatkini* occur in different habitats, (2) the nuclear 28S D3 marker separates *P. zachvatkini* from other members of its genus, and (3) morphological traits [detected by scanning electron microscopy (SEM) pictures] separate the two species *P. punctum* and *P. zachvatkini*.

## Material and methods

### Sampling

As part of a soil zoological survey within the project INPEDIV (‘Integrative analysis of the influence of pesticides and land use on biodiversity in Germany’), we took soil samples from the Eifel region in Germany in the federal states of North Rhine-Westphalia and Rhineland-Palatinate. Sampling took place at five study sites in May 2019 (RL1-RL5) and five different sites in May 2020 (RL6-RL10) (Table 1).

**Table 1:** Sampling sites in the German Eifel region

Code	Site name, nearest village	Sampling date	Coordinates
RL1	Tiesberg, Iversheim	14.5.2019	50°35′06.9″N, 6°45′45.8″E
RL2	Prachtacker, Ahrhütte	14.5.2019	50°23′33.0″N, 6°43′16.0″E
RL3	Baumberg, Wiesbaum	13.5.2019	50°20′39.4″N, 6°38′50.2″E
RL4	Eierberg, Alendorf	15.5.2019	50°22′01.3″N, 6°37′46.5″E
RL5	Kalvarienberg, Alendorf	15.5.2019	50°22′08.6″N, 6°38′27.4″E
RL6	Lambertsberg, Holzmühlheim	12.5.2020	50°33′28.0″N, 6°42′57.8″E
RL7	Halsberg, Gilsdorf	12.5.2020	50°33′04.2″N, 6°42′03.6″E
RL8	Jakop-Kneip-Berg, Gilsdorf	13.5.2020	50°32′36.5″N, 6°42′17.6″E
RL9	Auf Lind bei Esch, Esch	13.5.2020	50°22′03.4″N, 6°37′16.1″E
RL10	Mäuerchenberg, Gönnersdorf	13.5.2020	50°19′54.0″N, 6°42′57.8″E

All sites are nature reserves adjacent to agricultural fields

**Fig. 1:**
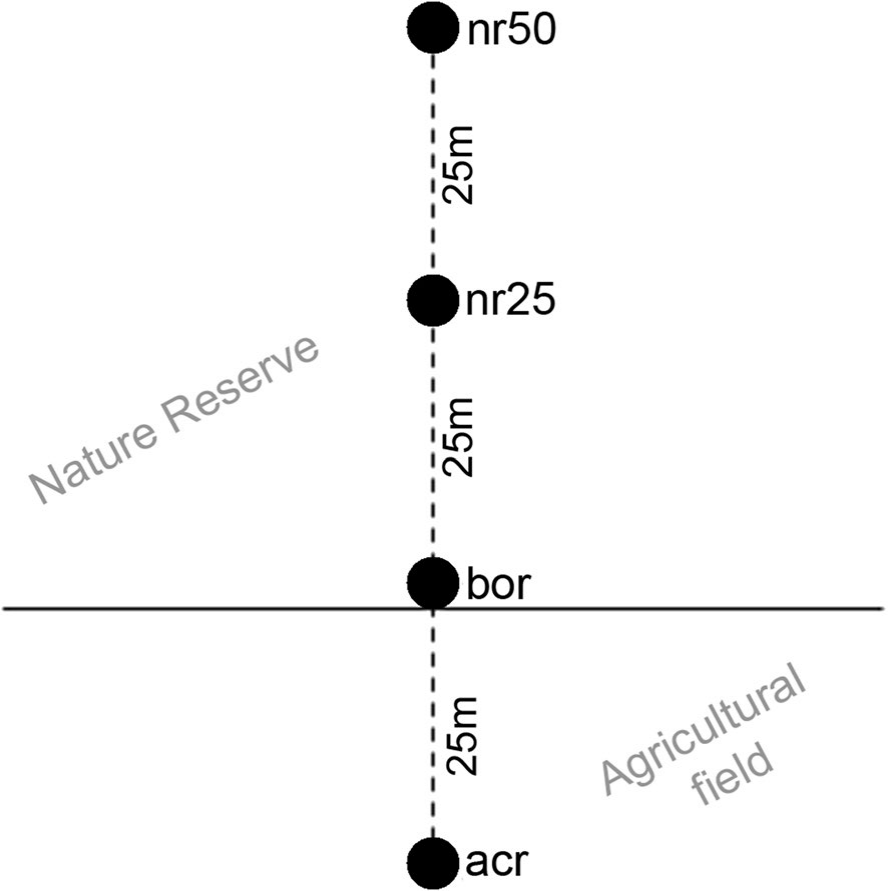
Sampling design along a transect from an agricultural field into a dry meadow in a nature reserve. Points mark plots at the agricultural field (acr), border (bor), 25 m inside the nature reserve (nr25) and 50 m inside the nature reserve (nr50)

At every site, we established a linear transect of four plots reaching from an agricultural field into an adjacent dry meadow located in a nature reserve. The first sampling plot was located 25 m within the agricultural field (plot ‘field’). The second sampling plot was located on the border between the agricultural field and the nature reserve (‘border’). The third and fourth sampling plot lay 25 m (‘nr25’) and 50 m (‘nr50’) apart from the border in the nature reserve (Fig. 1). At a distance of 4 m from the center of each plot, we took three soil samples with a metal cylinder (2.5 cm radius, 5 cm high), closed with lids on the top and bottom and cooled until extraction 2 or 3 days after field sampling. The samples were extracted over a 10-day period by a high-temperature gradient (from 20 °C on the first to 55 °C on the last day) using a MacFadyen apparatus (MacFadyen 1961), and preserved in 96% ethanol. We identified specimens of *P. punctum* and *P. zachvatkini* under the light microscope (Leica DM 2500) using the keys of Weigmann (2006), Shaldybina (1969), Bayartogtokh et al. (2000) and Seniczak et al. (2020). All specimens were deposited in the collection of the Senckenberg Museum of Natural History Görlitz (SMNG).

To test for correlations with soil parameters, we measured the water content, pH, nitrogen content, carbon content and particle size distribution separately for every soil sample. We calculated water content as weight loss per g of dry soil by weighing the soil samples before and after drying on the MacFadyen apparatus. We measured pH with a pH-meter model HI2210 (Hanna instruments, Vöhringen, Germany) in 1-M potassium chloride according to DIN ISO 10390 (DIN 2005). We performed measurements of carbon and nitrogen contents with 5–10 µg of dried and sieved sample soil in a vario PYRO cube elemental analyzer (Elementar Analysesysteme, Hanau, Germany). We measured particle size distribution as the proportion of clay (particle size < 2 µm), silt (2–200 µm) and sand (200–2000 µm) in each sample using a LS 13 320 particle size analyzer (Beckman Coulter, Miami, FL, USA).

For the genetic analyses, further specimens were provided within ‘MetaInvert’, a Senckenberg project in association with the ‘Forstliche Versuchs- und Forschungsanstalt Baden-Württemberg’. The MetaInvert specimens of *P. punctum* were sampled in 2018 at Olba Lake near Wartha (51°16′18.5″N, 14°36′36.8″E) and in Görlitz (51°07′56.6″N, 14°58′20.2″E), both in Saxony, Germany. Furthermore, we added one specimen of *Minunthozethes semirufus* (C.L. Koch) sampled in 2018 from the Sernitz bog (53°04′58.8″N, 13°55′08.4″E), Brandenburg, Germany, to the dataset as an outgroup, and downloaded additional sequences from GenBank (Table 2).

**Table 2:** Origin of 28S D3 sequences of four Punctoribatidae species used in the molecular analysis

Taxon	Seq	Locality	Source	GenBank number
*Punctoribates punctum* (C.L. Koch)	5	Tiesberg, field (Eifel)	Present study	OM699042–OM699046
	3	Baumberg, field (Eifel)	Present study	OM699047–OM699049
	2	Albersdorf, forest edge (SH)	GenBank (Lehmitz and Decker 2017)	KY686508, KY686509
	4	Wartha, Olba Lake (Sn)	MetaInvert	ON054028–ON054030, ON054032
	1	Görlitz, Loenscher Grund (Sn)	MetaInvert	ON054031
*Punctoribates zachvatkini* (Shaldybina)	1	Tiesberg, field (Eifel)	Present study	OM699050
	2	Prachtacker, nr50 (Eifel)	Present study	OM699051, OM699052
	2	Eierberg, nr25 (Eifel)	Present study	OM699053, OM699054
	1	Kalvarienberg, nr50 (Eifel)	Present study	OM699055
*Punctoribates sellnicki* (Willmann)	5	NSG Katenmoor (SH)	GenBank (Lehmitz and Decker 2017)	KY686510–KY686514
*Minunthozetes semirufus* (C.L. Koch)	3	Schafstedt, forest edge (SH)	GenBank (Lehmitz and Decker 2017)	KY681303–KY681305
	1	Sernitz bog (BB)	MetaInvert	ON054033

Sequences were either from specimens collected in the present study, during the MetaInvert project or downloaded from GenBank. *Seq* number of sequences, *SH* Schleswig–Holstein, *Sn* Saxony, *BB* Brandenburg (German Federal states)

### Habitat preferences

We calculated densities (number of individuals per m^2^) of both species for each sample from the field study and pooled samples according to plot (field, border, nr25, nr50). We found no specimens of *P. punctum* at three sites in 2020 (RL7, RL8, RL10) and excluded them from the analyses of *P. punctum*, leaving 84 samples from seven sites (RL1-RL6, RL9)*. Punctoribates zachvatkini* was present in all 10 sites, so we used all 120 samples for analyses of the species. We created boxplot diagrams of the average densities of the two species along the gradient from agricultural site into nature reserve using R v 4.0.4 (R Core Team 2021).

To extract ecological information from the museum material, we grouped sampling sites (if possible) into six categories depending on habitat type, namely: ‘grasslands’ for any kind of meadows including pastures; ‘agricultural sites’ for crop fields, plantations, intense pastures and vineyards; ‘coniferous forests’ and ‘deciduous/mixed forests’ for the respective forest types; ‘lake and river banks’ for floodplains, floating material at river banks and two peatland sites; and ‘anthropogenic’ for urban areas and former coal mining sites.

### Genetic distances

We extracted DNA non-destructively from 71 single whole individuals sampled in the Eifel region (see Sampling, above) using the Qiagen DNAeasy Blood&Tissue kit following the standard kit protocol. Specimens were incubated for 2–3 days in Qiagen Incubation lysis buffer (Lehmitz and Decker 2017) and the remaining body was transferred to 70% ethanol and stored in the voucher collection of the SMNG. Each specimen received an individual code consisting of the site where it was collected, the abbreviation of the plot, the number of the sample from that plot (1–3) and an individual letter for each individual of a sample (e.g., RL1-acr-3a = individual *a* from the *third* sample of the *agricultural* plot at *RL1*).

We used DNA extracts from 24 specimens (12 of each species) for DNA sequencing in both directions. We amplified the non-coding D3-D5 fragment of the 28S rDNA (and its flanking regions) of approximately 400–600 base pairs (bp) length using the forward primer D3A (5’-GACCCGTCTTGAAACACGGA-3’; Litvaitis et al. 1994) and the reverse primer 28Sbout (5’-CCCACAGCGCCAGTTCTGCTTACC-3’; Tully et al. 2006). The PCR reaction mix had a total volume of 10 µl, containing 0.12 µM of each Primer, 0.2 U Maximo Taq 1xl Taq buffer, 0.12 µM of each dNTP, and additive magnesium to a total of 2.0 mM (all components: GeneON, Ludwigshafen, Germany), 1 µl template DNA and 7.6 µl double-distilled water. PCR program ran with one pre-heating cycle at 95 °C for 60 s, followed by 33 amplification cycles (denaturation: 94 °C for 20 s, 49 °C for 20 s, and 68 °C for 45 s) and one post-annealing cycle at 72 °C for 10 min.

We inspected PCR products on a 1% agarose gel, purified single band amplifications with ExoSap-IT (Applied Biosystems) and sequenced them at the BiK-F Bio-diversity and Climate Research Centre, Frankfurt am Main, Germany. We checked all obtained sequences with BLAST in GenBank for contamination and manually aligned them in ClustalX v.1.83 (Chenna et al. 2003).

### Scanning electron microscopy (SEM)

We took SEM images of four specimens of *P. punctum* and five of *P. zachvatkini* from the field study (see Sampling, above). After dehydration of specimens in 96% alcohol, we critical-point-dried them in CO_2_ and mounted them on aluminum stubs before sputter-coating them with gold. We took images with a JEOL JSM-6510 LV microscope, which we edited using Adobe Photoshop CS4 for better contrast and discernment of fine structures.

### Distribution in Germany

To evaluate the distribution of *P. zachvatkini* and *P. punctum* in Germany, we re-identified all material of *P. punctum* and *P. zachvatkini* under the light microscope (Leica DM 2500) available from museum collections of SMNG (1841 specimens earlier identified as *P. punctum* and 4925 specimens earlier identified as *P. zachvatkini*), the Senckenberg Research Institute and Museum in Frankfurt (49 specimens earlier identified as *P. punctum*), the State Museum of Natural History in Karlsruhe (655 specimens earlier identified as *P. punctum*) and the Museum for Natural History in Berlin (486 specimens earlier identified as *P. punctum*). The material from the museum collections originated from Germany, Austria, Czech Republic and Slovakia.

We created a distribution map of *P. zachvatkini* in Germany with Edaphobase (www.portal.edaphobase.org), an online database for soil fauna (Burkhardt et al. 2014) and added further findings of *P. zachvatkini* from Weigmann et al. (2015), the field study in the Eifel region, and the museum material not included in Edaphobase using Adobe Photoshop CS4.

### Statistical analysis

#### Habitat preferences

We performed a two-way ANOVA in Past v.4.07b (Hammer et al. 2001) with the factors ‘plot’ and ‘site’ on each species independently to see whether the factors had a significant effect on density. For significant results, we performed Tukey’s post-hoc test to identify which sites/plots differed significantly (α = 0.05) from each other.

Furthermore, we tested for correlations between species abundances and the soil parameters water content, pH, nitrogen content, carbon content, and particle size distribution. As clay content was low in all samples (< 12%), the particle size distribution mostly differed in relation of silt to sand. We chose sand content as the parameter to represent particle size distribution. For each species and soil parameter, we calculated the Pearson correlation coefficients R^2^ in Microsoft Excel. We used all samples in these analyses.

To see if combinations of parameters affected dominance, we created contour diagrams of both species and each of the 10 possible parameter combinations in R using R packages ggplot2 3.3.5 (Wickham 2016) and contourPlot v0.2.0 (Murphy 2020). A species was considered dominant when it was more abundant in a sample compared to the other species. We excluded samples with equal densities in both species from the analyses, leaving 64 samples. We considered species to differ in dominance when the contour plots of the relevant parameters did not overlap.

#### Genetic distances

There was no variation within the D5 fragment of *Punctoribates* in our dataset, so we only used the D3 fragment (315 bp) for analyses as most GenBank sequences only cover this region. We selected eight high-quality sequences of *P. punctum* and six of *P. zachvatkini* for analyses, and added additional sequences from GenBank of *P. punctum* as well as *Punctoribates sellnicki* (Willmann), a species with porose areas, to the dataset. Furthermore, we added sequences of *P. punctum* and *Minunthozetes semirufus* (C.L. Koch) obtained during the MetaInvert project (see Sampling, above). In total, the dataset consisted of 30 sequences from four species of the family Punctoribatidae (Table 2).

Sequences were aligned with MUSCLE and modified in MEGA6. To test for species separation, we calculated pairwise distances (p-distances) as the number of differences per base position between sequences, and created a Neighbor-Joining tree (Saitou and Nei 1987) to see if species formed separate clades. The optimal tree with the sum of branch length = 0.10 is shown. The tree is drawn to scale, with branch lengths in the same units as those of the evolutionary distances used to infer the phylogenetic tree. The evolutionary distances were computed using the p-distances method (Nei and Kumar 2000) and are in the units of the number of base differences per site. There were in total 316 positions in the final dataset. For p-distances and the tree, all ambiguous positions were removed for each sequence pair. All evolutionary analyses were conducted in MEGA6 (Tamura et al. 2013).

## Results

### Habitat preferences

**Fig. 2:**
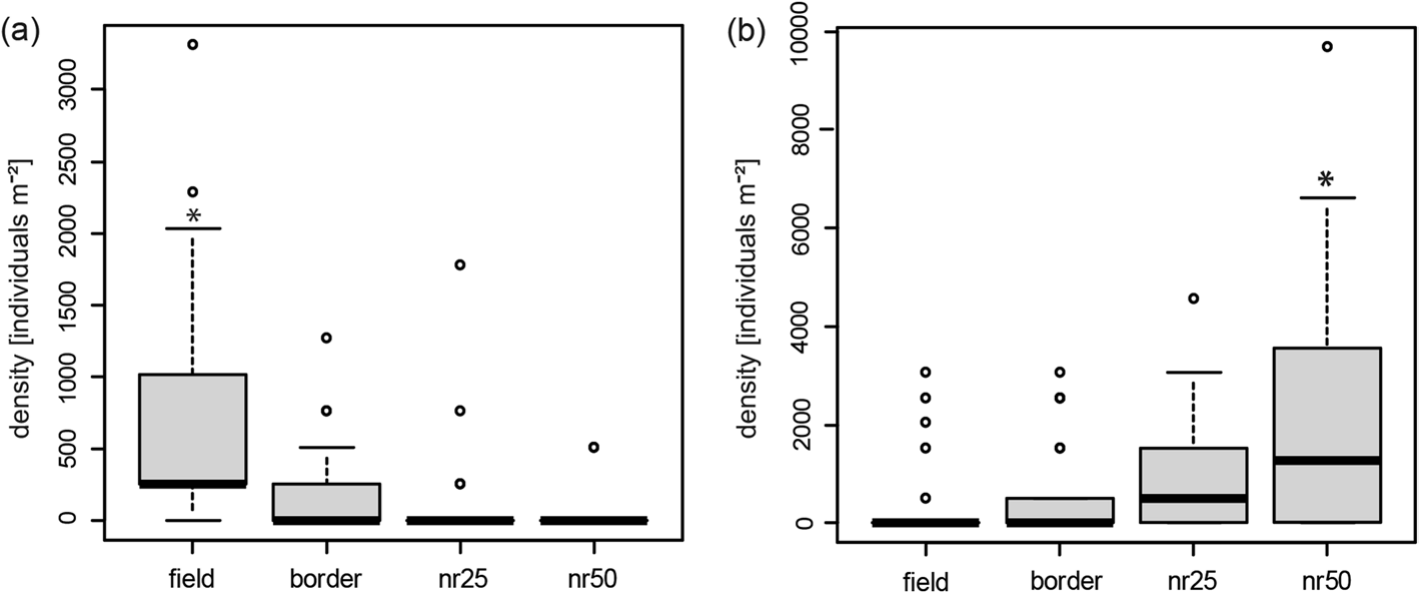
Densities (no. individuals m^−2^) of **a** *Punctoribates punctum* (n = 21) and **b** *P. zachvatkini* (n = 30) along the gradient from agricultural sites into nature reserves. Boxes represent 50% of variation, thick lines inside the boxes indicate the median, and whiskers 1.5 × the interquartile range, with dots marking outliers. Plots significantly different from others according to Tukey’s post-hoc test are marked with an asterisk. nr25 = plot 25 m inside the nature reserve, nr50 = plot 50 m inside the nature reserve

Overall, we found 99 specimens of *P. punctum* and 209 specimens of *P. zachvatkini* in the Eifel region. Densities differed between sites (2-way ANOVA, *P. punctum*: F_6, 56_ = 3.07, P = 0.011; *P. zachvatkini*: F_9, 80_ = 2.14, P = 0.036) and along the gradient from the agricultural fields into the nature reserves (*P. punctum*: F_3, 56_ = 10.35; *P. zachvatkini*: F_3, 80_ = 10.84, both P < 0.001). There was no significant interaction between site and plot. Densities of *P. punctum* were higher in the field compared to other plots (Tukey’s test: field vs. border: P = 0.001; field vs. nr25/nr50: P < 0.001), whereas densities of *P. zachvatkini* were higher in nr50 plots (nr50 vs. field/border: P < 0.001; nr50 vs. nr25: P < 0.05) (Fig. 2).

**Fig. 3:**
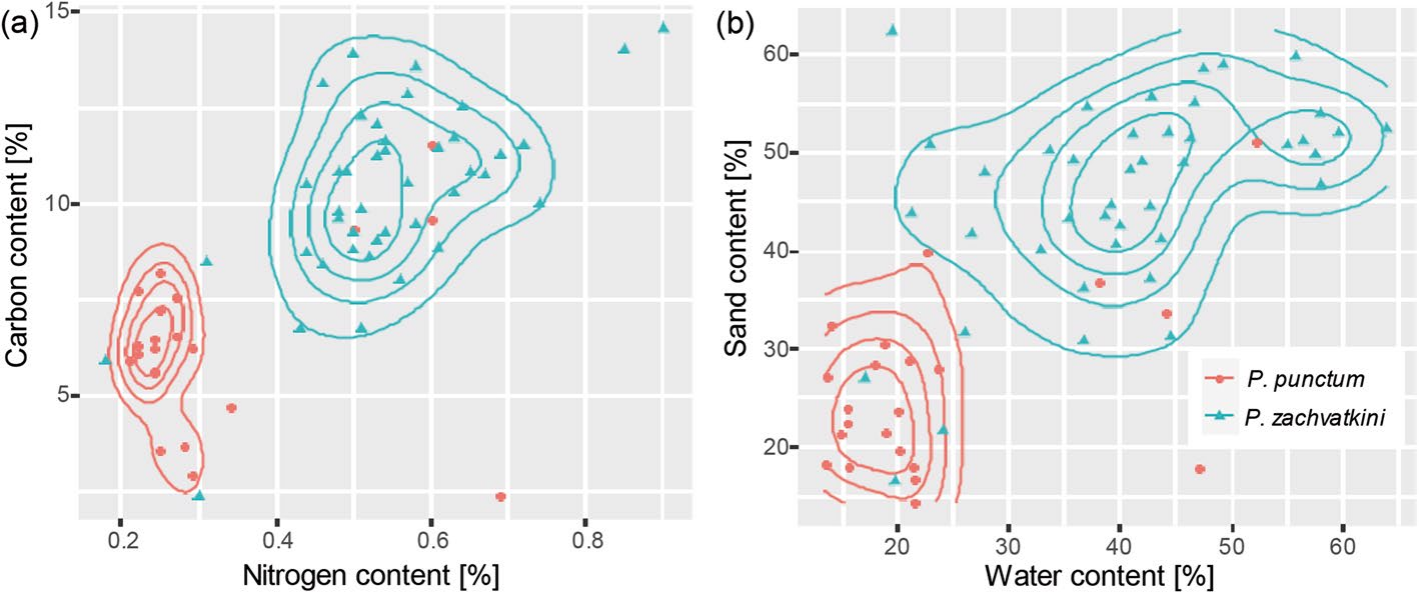
Contour plots showing ecological range of dominance for **a** combination of soil carbon and nitrogen content and **b** soil water and sand content (%). For samples where both *Punctoribates punctum* and *P. zachvatkini* co-occurred, only the dominant species (higher density) was mapped

No single abiotic parameter explained the distribution of either species (Pearson correlation, R^2^ < 0.2). Contour plots showed that *P. zachvatkini* was dominant in nutrient-rich and *P. punctum* in nutrient-poor soils (Fig. 3a). *Punctoribates zachvatkini* also dominated in moist and sandy soils (Fig. 3b).

**Fig. 4:**
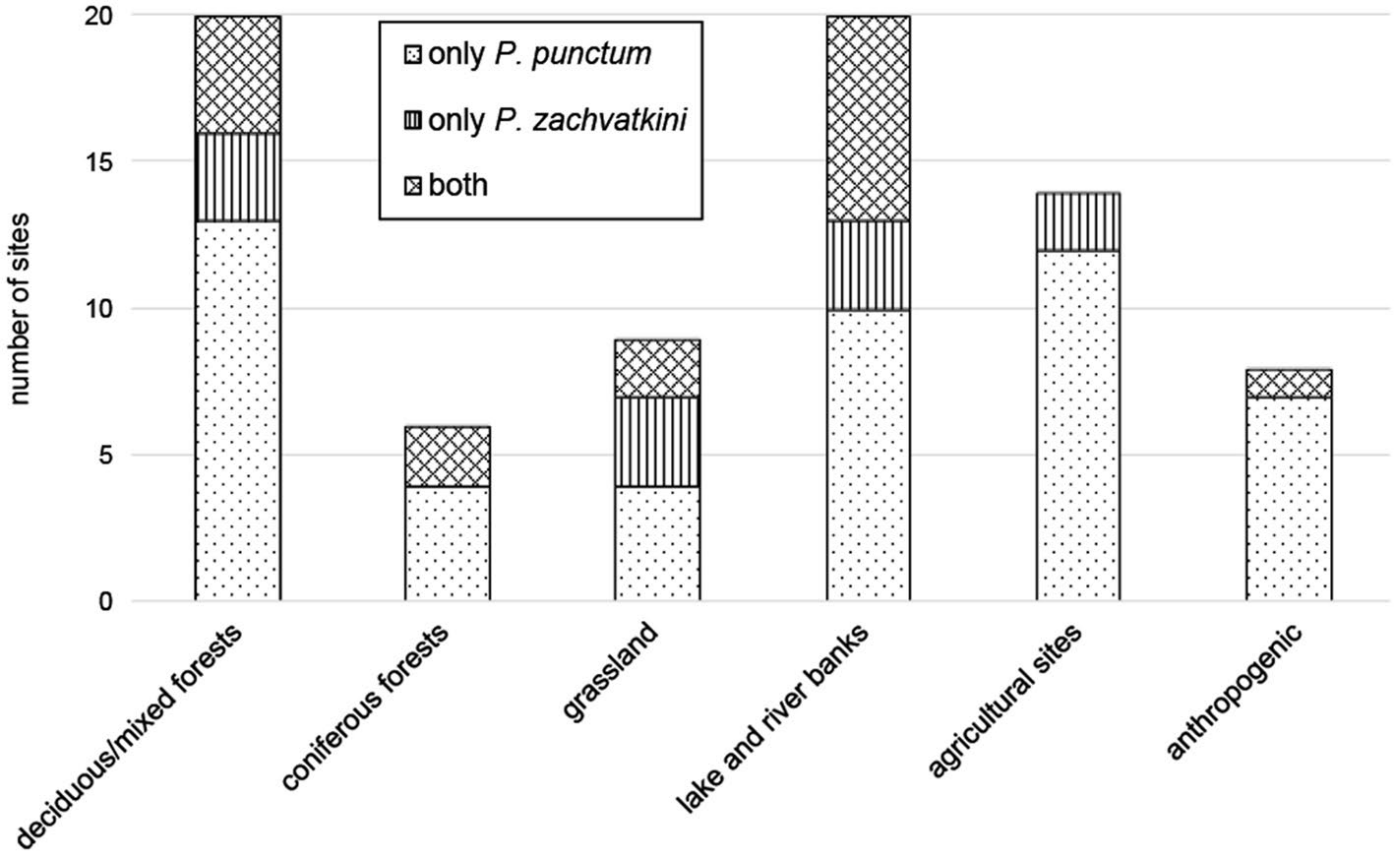
Number of sampling sites represented in the museum collections by 77 sampling events of either only *Punctoribates punctum*, only *P. zachvatkini* or both species together, sorted by different habitat types

Regarding the material from museum collections, we found more sampling sites were colonized by *P. punctum* than by *P. zachvatkini* or both species together. However, *P. zachvatkini* also occurred in almost all habitat types, including lake and river banks, forests and grasslands. *Punctoribates punctum* was the more common species in highly disturbed anthropogenic habitats (cities, post-mining sites) and on agricultural sites, whereas *P. zachvatkini* was rare (Fig. 4).

### Genetic distances

**Fig. 5:**
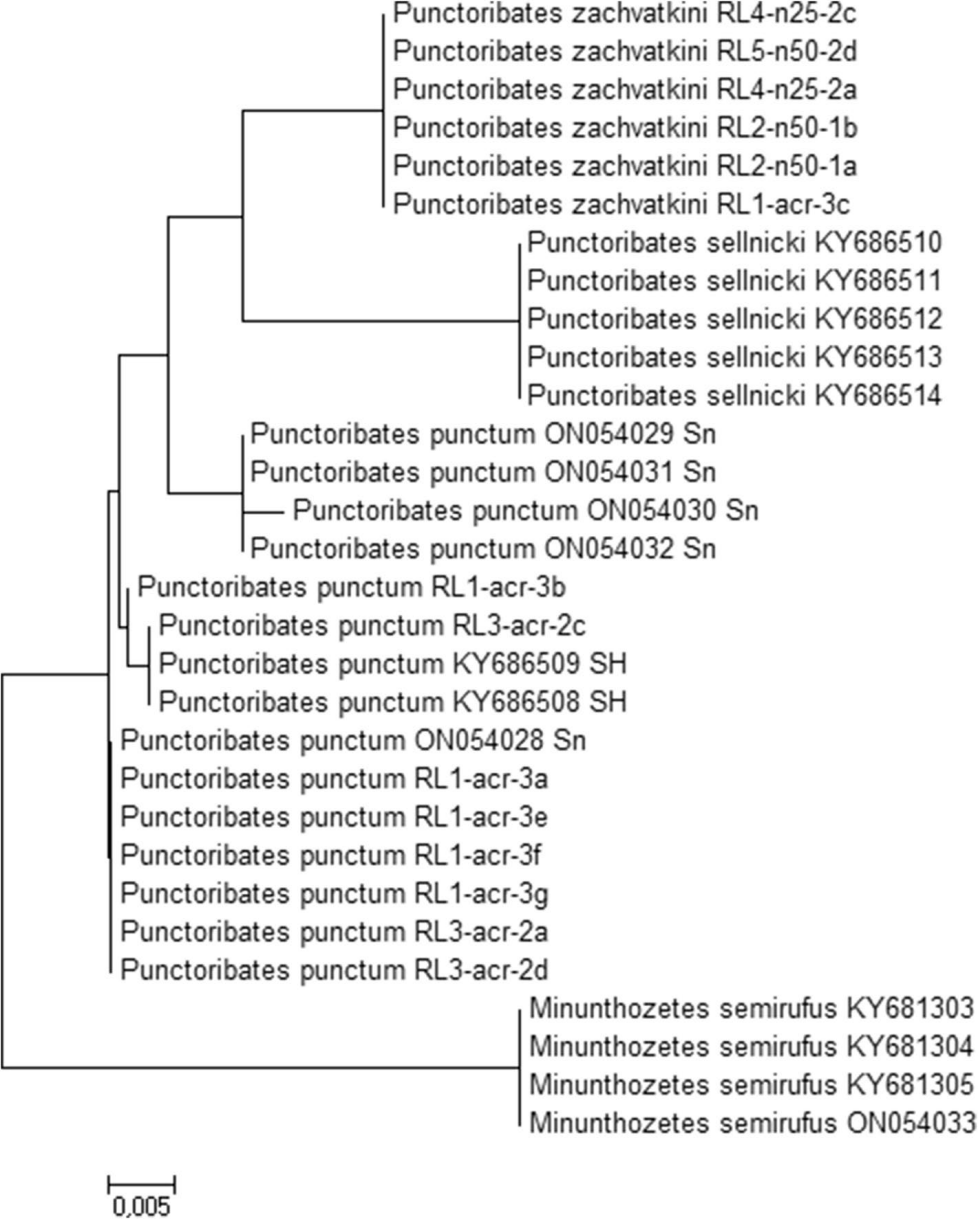
Neighbor-Joining tree based on sequences of the D3 fragment of 28 s rDNA. The tree is drawn to scale, with branch lengths in the same units as those of the evolutionary distances used to infer the phylogenetic tree. Names of Eifel specimens consist of site, plot, sample number of the plot and a letter individual to each specimen of a sample (see text for details). *Sn *specimen from Saxony, *SH* specimen from Schleswig–Holstein

Of the 316 base positions, 28 showed differences between the four species. Between *P. punctum* and *P. zachvatkini* p-distances of the D3 fragment of 28S rDNA ranged from 1.9 to 2.9%. Specifically, *P. zachvatkini* contained 5–8 substitutions compared to *P. punctum*. Among sequences of *P. punctum* from the Eifel region p-distances varied by 0–0.3%, whereas there was no variation within the sequences of *P. zachvatkini*. Comparing sequences of *P. punctum* among regions, those from the Eifel region differed by 0–1.6% from those of other regions: by up to 0.3% from specimens from Schleswig–Holstein and by up to 1.6% from specimens from Saxony. *P. sellnicki* and *P. punctum* differed by 2.9–3.8%, *P. zachvatkini* and *P. sellnicki* by 3.2%. To *Minunthuzetes semirufus*, the *Punctoribates* species differed by 4.8–7.6% (Table 3). All species formed separate clades in the Neighbor-Joining tree (Fig. 5).

**Table 3:** Pairwise distances between *Punctoribates zachvatkini* and *P. punctum* sequenced in the present study and the oribatid mite species from the dataset (origin see Table 2)

Species A	Species B	p-distances [%]
*Punctoribates punctum* Eifel	*P. punctum* Eifel	0.0–0.3
	*P. punctum* SH	0.0–0.3
	*P. punctum* Sn	0.0–1.6
	*P. zachvatkini*	1.9–2.2
	*P. sellnicki*	2.9–3.2
	*Minunthozetes semirufus*	4.8–5.1
*Punctoribates zachvatkini*	*P. zachvatkini*	0.0
	*P. punctum* SH	1.9
	*P. punctum* Sn	1.9–2.2
	*P. sellnicki*	3.2
	*Minunthozetes semirufus*	7.0

*Sn* specimen from Saxony, *SH* specimen from Schleswig–Holstein

Alongside the differences in p-distances, all six individuals of *P. zachvatkini* from the various study sites had an additional base pair at position 101 in the D3 region of 28S compared to the other Punctoribatidae species analyzed in this dataset.

### Scanning electron microscopy

**Fig. 6:**
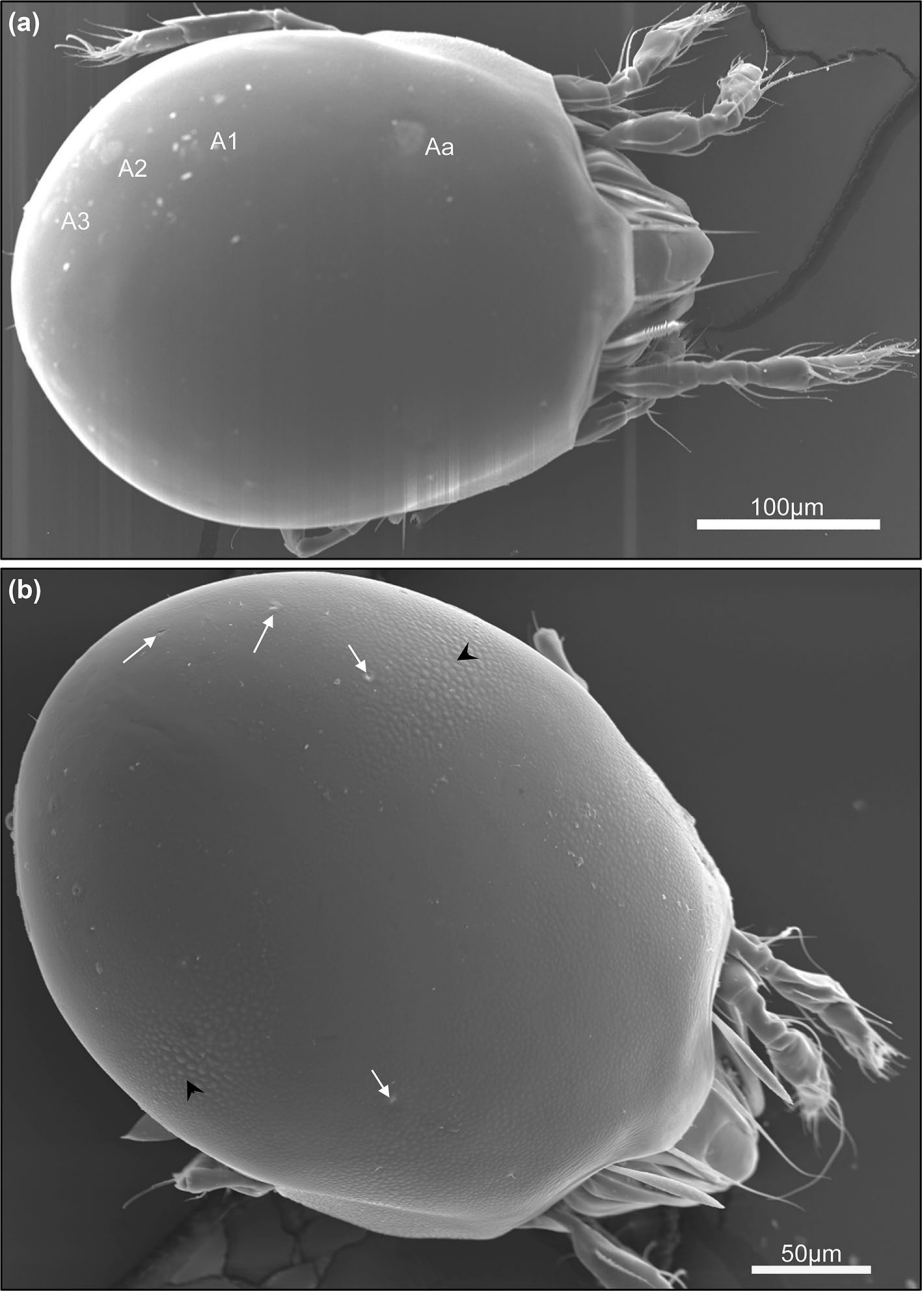
Scanning electron microscopy overviews of **a** *Punctoribates punctum* and **b** *P. zachvatkini*, dorsal view. *Aa* anterior porose area, A1-3 = posterior porose areas 1–3; white arrows point at saccules, black arrowheads at regions of large cuticular protrusions

**Fig. 7:**
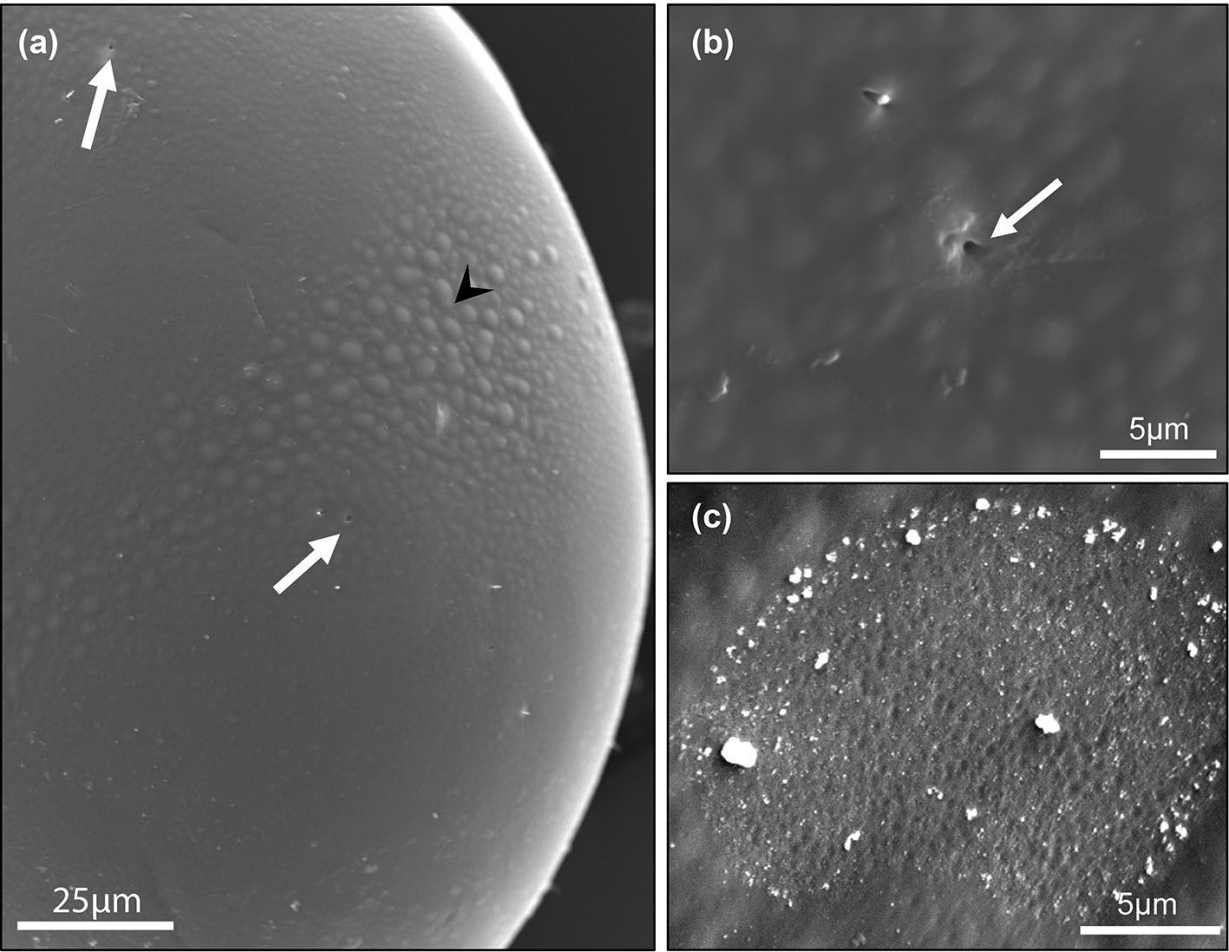
Scanning electron microscopy images of the octotaxic system of (**a**, **b**) *Punctoribates zachvatkini* and (**c**) *P. punctum*, dorsal view. **a** Lateral posterior part of notogaster of *P. zachvatkini*; **b** close-up on saccule of *P. zachvatkini*; **c** close-up on porose area of *P. punctum*. White arrows point at saccules, black arrowhead points at region of large cuticular protrusions

The SEM images of the four specimens of *P. punctum* and five specimens of *P. zachvatkini* showed the different expressions of the octotaxic system of both species in fine detail (Figs. 6, 7): four pairs of porose areas or saccules, respectively, one pair in the anterior half of the notogaster at the height of the posterior end of the pteromorphs, and three pairs near the posterior-lateral margin of the notogaster. The porose areas of *P. punctum* (Fig. 6a, 7c) were largest in the anterior pair (Aa) while smaller in the posterior ones (A1-3). The saccules of *P. zachvatkini* (Figs. 6b, 7a,b) were visible as small openings, all of similar size, in the notogaster.

The investigated specimens showed another fine structure that is different between the two species: they both had an uneven surface with small cuticular protrusions on the notogaster, but in *P. zachvatkini* these protrusions were noticeably larger near the central-lateral areas of the notogaster (Figs. 6b, 7a, arrow heads). The cuticle of *P. punctum* on the other hand was similar in this region to the rest of the body (Fig. 6a).

### Distribution in Germany

**Fig. 8:**
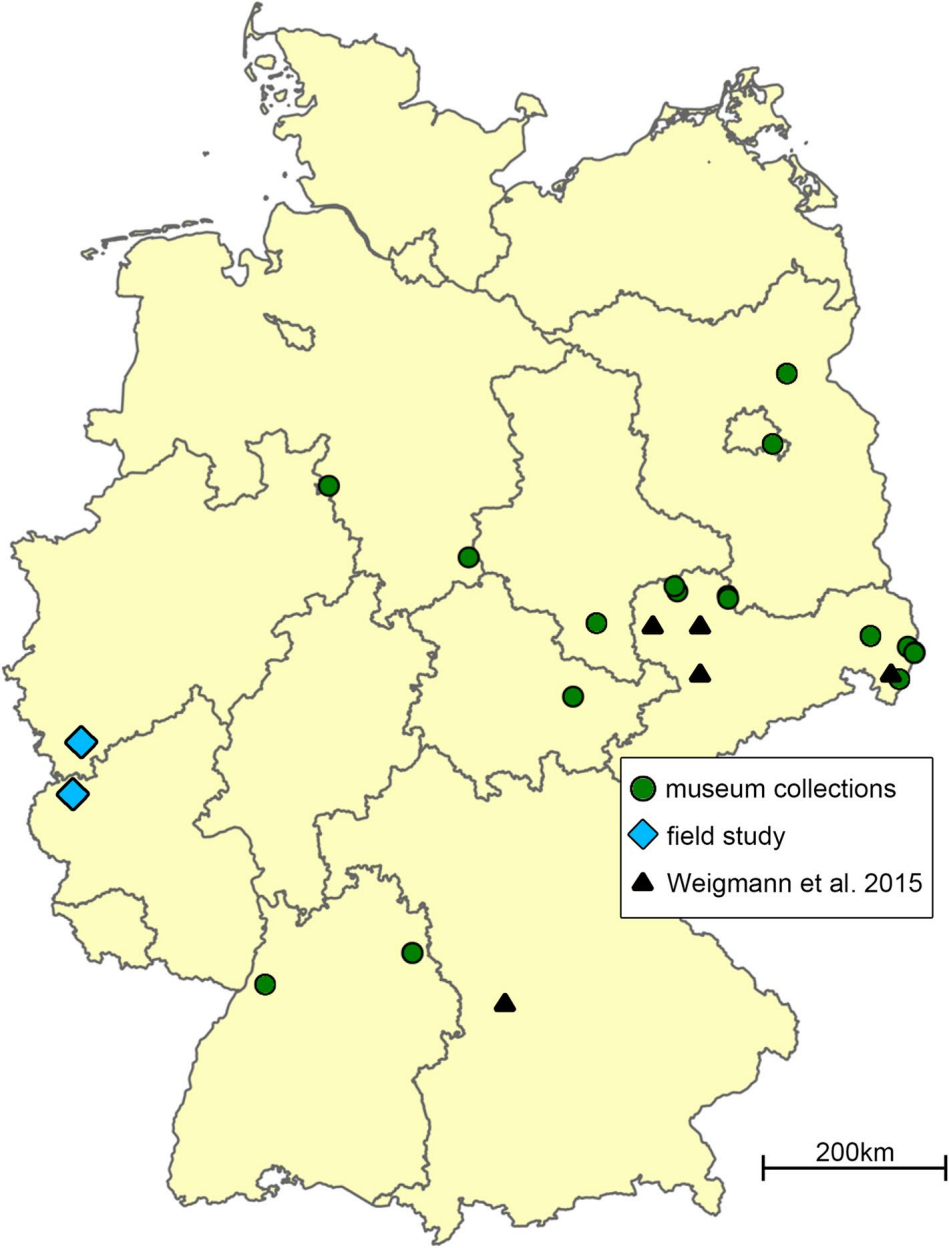
Findings of *Punctoribates zachvatkini* in Germany from museum collections after review, the field study in the Eifel region and Weigmann et al. (2015). Map created in Edaphobase (www.portal.edaphobase.org) and edited with Adobe Photoshop CS4

We identified 5259 specimens of *P. zachvatkini* and 2687 specimens of *P. punctum* from the museum collections. We re-identified 351 specimens of *P. punctum* as *P. zachvatkini*, and seven specimens of *P. zachvatkini* as *P. punctum*. The earliest record of *P. zachvatkini* in Germany stemmed from 1967 from a chalk slope near Mücheln, Thuringia. The 41 individuals of this sample were collected by Manfred Moritz and are stored in the collection of Jason Dunlop at the Museum of Natural History in Berlin. The most western distribution of *P. zachvatkini* in Europe stemmed from the field study in the Eifel region, at site RL10 near Gönnersdorf (Fig. 8).

## Discussion

*Punctoribates punctum* and *P. zachvatkini* have mainly been distinguished by the type of their octotaxic system, a trait that may be variable within other species of Poronota (Weigmann 2010; Weigmann and Ermilov 2016). The present study shows that these two species also differ in regard to their habitat requirements, in regard to the sequence of the genetic species marker 28S D3, and in regard to the fine protrusions on their notogaster. These findings support their status as two separate species.

Intraspecific variation of the D3 fragment of 28S rDNA in Oribatida usually neither exceeds 0.5% nor includes indels (Lehmitz and Decker 2017). We found both to be the case when comparing sequences of *P. punctum* and *P. zachvatkini*, with all specimens of *P. zachvatkini* having at least five substitutions and an additional base pair. In accordance with the p-distances, the neighbor joining tree splits the species into different clades. Variation within *P. punctum* from different geographical regions exceeded 0.5%. Specifically, some sequences from Saxony differ by 1% or more from individuals of other regions. Examination of the corresponding vouchers confirmed this variation is neither due to contamination nor misidentification, and the sequences were not ambiguous. Further studies are needed to clarify whether *P. punctum* is possibly a complex of cryptic species and whether the genetic variation is also reflected morphologically. As this is the first genetic analysis conducted on *P. zachvatkini*, so far no information on genetic variance between populations is available for comparison.

There was no correlation of the abundance of *P. zachvatkini* or *P. punctum* with single soil parameters. According to Acarofauna Germanica published by Weigmann et al. (2015), *P. zachvatkini* is associated with wet or moist habitats such as fresh meadows or alluvial forests (in Germany). *Punctoribates zachvatkini* from the field study and museum material was present in other habitats as well, such as dry meadows and coniferous forests. *Punctoribates punctum* avoids low pH (Wissuwa et al. 2013). As the pH values of the study sites ranged from 5.5 to 8.1, they may have not been acidic enough to verify this effect. Instead, we found that combinations of soil parameters affected distribution of the two species, with *P. zachvatkini* being more dominant in nutrient-rich, wet and sandy soils. The observed niche ranges match the generalist ecology of *P. punctum*, which is able to colonize habitats outside the range of *P. zachvatkini*.

Parameters relating to agricultural practices such as pesticide application or ploughing may also play a role. In the field study in the German Eifel region, *P. zachvatkini* had higher densities in the plots furthest away from the field in the protected dry meadows of the nature reserves, whereas densities of *P. punctum* were highest in the agricultural plots. This is in line with earlier research which found *P. punctum* to be an early colonizer of disturbed habitats, including agricultural sites (Sheals 1956; Murvanidze et al. 2013; Wissuwa et al. 2013), and usually more often present at sites of anthropogenic influence compared to *P. zachvatkini* (Ivan and Călugăr 2004; Kolodochka and Shevchenko 2013; Shevchenko and Kolodochka 2014). In the museum material, *P. punctum* was also more common in highly disturbed habitats such as urban areas or post mining sites than *P. zachvatkini*. Their opposing distributions might make these species good indicators for anthropogenic disturbances of soil fauna.

The observed difference in habitat preference can be the result of a competitor-colonizer trade-off, where one species is a better competitor in regard to a resource while the other is better at colonizing habitats (Kneitel and Chase 2004). *Punctoribates punctum* can be transported by wind and water (Schuppenhauer et al. 2019) and accordingly, might be able to recolonize a habitat more quickly after disturbance than can *P. zachvatkini*. In contrast, *P. zachvatkini* may have a competitive advantage over *P. punctum* in less disturbed areas. Taken together this would explain our present findings.

Differences in the habitat preferences and type of octotaxic system of the two species might be linked. Little is known about how the type and function of the octotaxic system are related to the ecological demands of the animals. Alberti and Norton (1997) suggested invagination of open pore fields, meaning expression of saccules, might minimize water loss across the cuticle, but found no correlation with desiccating environments. In contrast, they found that secretory porose areas are often enlarged in species associated with dry environments. This is in line with the findings of the present field study where *P. punctum* with open porose areas was more abundant in the generally dryer (agricultural) sites.

In addition to having different octotaxic systems, *P. zachvatkini* is seen in SEM images to have larger cuticular protrusions in certain regions of the notogaster compared to *P. punctum*. In the original description, Shaldybina (1969) described *P. zachvatkini* with dark punctulation on the integument. The protrusions seen in the SEM images are probably identical to this punctulation. Though Shaldybina describes the punctulation to be dark and noticeable, the protrusions seem rather inconspicuous under the light microscope and share the same color as the notogaster. They only appear dark and striking in individuals that have been intensively cleared in lactic acid or in voucher specimens after DNA extraction. Ultimately, the octotaxic system is a better trait to morphologically distinguish *P. zachvatkini* from *P. punctum*.

A comprehensive review of collection material from natural history museums has shown that due to the morphological similarity, *P. punctum* and *P. zachvatkini* were insufficiently separated in the past, at least in Central and Western Europe. Before this study, the only German findings of *P. zachvatkini* stemmed from Saxony and Bavaria (Weigmann et al. 2015), and were considered its most western distribution. The present study extends its distribution to 10 of the 16 German federal states, covering the majority of the country from east to west. Its distribution might well expand further to the west into the neighbouring countries such as France, Belgium and Luxembourg. The earliest known record of *P. zachvatkini* in Germany now dates back to 1967, 2 years before Shaldybina (1969) described the species. This highlights the value of museum collections, which allow earlier findings to be reviewed in the light of the latest knowledge.

## Conclusion

The present investigation supports the status of *P. punctum* and *P. zachvatkini* as separate species*.* They are morphologically distinct beyond the expression of their octotaxic system and show clear differences in their habitat preferences as well as with respect to the nuclear 28S rDNA genetic marker. Although the octotaxic system may be labile in families and genera and can in few cases vary within populations of the same species, it appears to be reliable in separating species of *Punctoribates*. In addition, the present examination of museum material revealed an insufficient distinction between the two species in the past. With increasing knowledge about species and their distribution, re-evaluation of old findings and the storage of individuals in museum collections for future research is an important aspect of scientific work.

## Data Availability

Data on the distribution of *Punctoribates punctum* and *P. zachvatkini* in the Eifel region will be uploaded to Edaphobase, an open-access data warehouse on soil biodiversity (www.edaphobase.org), alongside the data from the review of the museum material. DNA sequences are available at GenBank (www.ncbi.nlm.nih.gov/genbank/, see Table 2 for access numbers).
